# Introduction of Micro-Scale CFD Model of Foam Injection Moulding Process

**DOI:** 10.3390/polym18121433

**Published:** 2026-06-08

**Authors:** Daniel C. Fritsche, Malte Schön, Christian Hopmann

**Affiliations:** 1 Chair of Plastics Processing, Rheinisch-Westfälische Technische Hochschule Aachen University, Seffenter Weg 201, 52074 Aachen, Germany; 2 3M Deutschland GmbH, Carl-Schurz-Straße 1, 41453 Neuss, Germany; mschoen@mmm.com

**Keywords:** foam injection moulding (FIM), microcellular foaming, OpenFOAM, volume of fluid (VoF), classical nucleation theory (CNT), multiscale simulation, bubble deformation, coalescence, cell growth kinetics

## Abstract

Foam injection moulding (FIM) enables lightweight thermoplastic parts, but current process simulations do not resolve microstructure formation. This work presents a micro-scale CFD framework for FIM that captures gas–melt interaction and bubble morphology. A two-phase, compressible volume-of-fluid solver (OpenFOAM) with surface tension and viscoelastic Phan–Thien–Tanner rheology is coupled to a nucleation pre-processor based on classical nucleation theory, which places bubbles stochastically using macro-scale pressure and temperature histories. The approach was demonstrated on a plate geometry using a 2D through-thickness section to investigate bubble nucleation, deformation, coalescence, and interaction under realistic process conditions. The simulations reproduced characteristic morphology trends across the thickness. In particular, the predicted aspect ratio and orientation show the expected skin–core behaviour and agree qualitatively with experimental observations. These results demonstrate that the framework can describe morphology development beyond simplified spherical-cell assumptions and provides a proof of concept for multiscale coupling between macro-scale process conditions and micro-scale foam structure evolution. A simplified surrogate growth representation was used to enable bubble expansion; however, a physically based mass-transfer model is required for quantitatively accurate growth kinetics.

## 1. Introduction

The thermoplastic foam injection moulding (FIM) process is widely used for the production of parts in the automotive, packaging and leisure industries. Physically foamed thermoplastic injection moulded components have many advantages over compact components. The foam structure allows for significant weight reduction while maintaining the same volume. Furthermore, the pressure of the blowing agents in the bubbles reduces shrinkage and warping. In the context of sustainability and ever-increasing technical requirements, the demand for foamed plastic components is therefore rising continuously [1,2].

Most commercial injection moulding simulation software still represents the foam microstructure only in highly simplified ways, with spherical/isotropic cells, fixed diffusion and without shear-induced deformation, coalescence, or collapse. The only results are the average cell radius and cell density. So, unlike compact moulding where simulation is accurate enough to display lab experiments, reliable prediction for foamed parts remains limited. The deficit becomes more severe in complex geometries because local pressure–temperature–shear histories differ strongly across the cavity, which affects the local microstructure [3]. However, the full potential of foamed components can only be exploited if the spatially inhomogeneous anisotropic foam structure and thus the mechanical and thermal properties of the components can be accurately predicted and designed to suit the specific application [4,5].

Modelling and simulation of the foam formation process have therefore been the focus of research for many years. For example, Chu and Xu investigated nucleation and cell growth in a CO_2_-loaded melt of polymethyl methacrylate using molecular dynamics simulations. They concluded that the bubble size distribution becomes increasingly homogeneous as the process pressure rises [6]. Complementary macro-to-micro-evidence shows that the final cell size distribution and morphology also depend on viscoelastic growth and depletion effects. Direct measurements combined with 3D simulations demonstrate how process pressure histories control elastic energy storage and, hence, the final cellular structure [7].

Park et al. investigated the solubility of physical blowing agents, such as CO_2_, in polymers. Solubility was determined using a modified pvT measuring device for linear and branched polypropylene. It was found that the solubility of physical blowing agents in polymer melts can be increased with increasing process pressure and decreasing temperature. The results were also compared with semi-empirical equations of state for predicting solubility. The potential for improvement in the equations of state for prediction was pointed out [8].

The research focuses in particular on cell nucleation and cell growth. Both macro-scale and micro-scale analyses have been carried out with regard to nucleation. Leung et al. investigated the influence of nucleating agents (e.g., talc) on the heterogeneous nucleation of CO_2_-loaded polystyrene melts. Bubble growth was visualised in situ using a special experimental setup. It was shown that the use of nucleating agents is accompanied by local pressure fluctuations, which promotes the nucleation of new cells. A comparison with the nucleation simulation by Wang et al. showed good agreement with the experiment [9,10,11].

On the micro-scale, several numerical simulation models have been developed and investigated that describe the nucleation, growth and solidification of cells, taking into account different boundary conditions. Arefmanesh and Advani developed a numerical approach called the ‘Cell Model’ to describe cell growth under non-isothermal boundary conditions and low process pressures. The numerical model was compared with experiments in terms of the bubble density (bubbles/cm^3^) and gas concentration. There were significant deviations between the experiment and the model, which can be attributed to the many simplifications [12].

Taki used a numerical approach to describe the nucleation and growth of spherical cells and compared this with experimental investigations. For constant pressure release rates, good agreement with the experimental results was achieved [13].

Ataei et al. used a modified Lattice Boltzmann Method (LBM) to describe cell growth at the micro-scale level. Here, the two-phase (liquid–gas) problem was simplified to one phase (liquid) and a comparatively low process pressure was used (15 MPa). The cell growth model is based on the ‘cell model’, which involves several simplifications: no interaction between neighbouring cells, incompressible melt, isothermal bubbles, constant diffusion rates, etc. However, the model is capable of performing a 2D or 3D simulation of the foaming process for specified boundary conditions. The model was then compared with the ‘Cell Model’ by Arefmanesh and Advani. A comparison of the simulative and experimental results with regard to the cell structure was not carried out [14,15].

Within the scope of commercially available simulation tools, modelling based on a homogeneous nucleation theory and the simplified modelling of cell growth is the state of the art [3,16]. The bubble geometry is assumed to be spherical and is not dependent on the flow behaviour of the plastic melt [17]. Shear effects, coalescence, bubble collapse, etc., are not taken into account. Due to locally inhomogeneous process variables resulting from high pressure variations, thermal processes and complex flow processes, such simplifications (symmetrical bubble geometry, constant diffusion rates) are not permissible for precise modelling.

The widespread adoption of FIM is hindered by the insufficient ability to predict the development of the cell structure for a specific geometry with sufficient accuracy, taking into account the local flow and solidification behaviour. Wang et al. calculated the weight reduction of complex foamed moulded parts using commercial simulation tools and found good agreement with experimental results [17]. However, since local cell orientation or coalescence are not taken into account, the calculated effective properties are insufficient for accurate component design. The result of the structural simulation is highly dependent on the predictive accuracy of the injection moulding process simulation [16,17].

Therefore, a multi-scale simulation approach is described to calculate the pressure, temperature and gas concentration on a macro-scale and simulate the gas cell geometry on a micro-scale [18]. The present work focuses on the development of a micro-scale simulation model in OpenFOAM that resolves bubble nucleation, deformation, coalescence, and gas–melt interactions based on macro-scale process conditions. To enable bubble expansion within the current solver framework, a simplified surrogate growth representation is employed, in which diffusion-driven mass transfer is not explicitly resolved. This approximation was used to study the qualitative behaviour of bubble–flow interaction and to establish a proof of concept for multiscale coupling. The model was subsequently used to analyse the phenomenological deformation of cells under characteristic injection moulding flow conditions.

## 2. CFD Model for Micro-Scale FIM

### 2.1. Software, Solver and Numerics

All simulations were performed with OpenFOAM v11 (OpenFOAM Foundation, London UK). The two-phase, compressible, immiscible flow was modelled with the solver compressibleVoF, which employs a volume-of-fluid (VoF) phase-fraction formulation with bounded-phase transport (MULES) and an interface compression term to maintain a sharp interface. Pressure–velocity coupling followed the PIMPLE algorithm (outer and inner correctors), and surface tension was represented by the Continuum Surface Force (CSF) model.

Because quasi-static effects (buoyancy, hydrostatics) are negligible in the present micro-scale model, gravity was disabled (g = (0,0,0)) [19]. This assumption can be justified by considering the Bond number, which compares buoyancy and surface tension forces. Using conservative estimates with a characteristic bubble size of up to 300 µm, a maximum density difference of Δρ ≈ 1200 kg/m^3^, and a minimum surface tension of σ = 0.0178 N/m, the Bond number is approximated as Bo ≈ 0.06. For more typical conditions (L~100 µm), the Bo is on the order of 10^−3^. Since Bo ≪ 1, surface tension forces clearly dominate over buoyancy, and gravitational effects can be neglected.

### 2.2. Governing Equations

The solver advances a single momentum equation for the homogeneous mixture, a phase fraction equation for the gas volume fraction (φ) (with MULES bounding and explicit interface compression), and an energy equation for non-isothermal conditions. Interfacial momentum exchange is modelled through the CSF surface tension force:

f_σ_ = σ κ ∇φ (1)

with the curvature (κ) obtained from the VoF field. Compressibility is handled with the thermodynamic packages of OpenFOAM for each phase (Section 2.4). Full derivations and implementation details of compressibleVoF and MULES can be found in the OpenFOAM sources and documentation [20,21,22].

### 2.3. Geometry and Mesh

The developed model considers the often-neglected wall thickness of injection-moulded parts. In foam injection moulding, cell nucleation and growth vary strongly across the thickness because temperature and shear/velocity gradients control the nucleation rate, cell deformation and coalescence. The goal is to describe the conventional skin–core morphology and property gradients with this model [23,24,25,26].

Therefore, the thickness and flow length are created as a 2D infinite-width model, as the velocity, temperature and pressure in the width direction are assumed to be negligible for a uniform flow front. We adopt this rationale at the micro-model scale to focus computational effort on the evolution that governs gas–melt segregation and the local pressure/temperature histories.

The computational domain is a straight channel with a length (L) of 4.0 mm and a thickness (Th) of 3.0 mm. A structured mesh was generated with blockMesh using uniform spacings, ΔT = 0.015 mm and ΔL = 0.02 mm, yielding 200 × 200 × 1 cells. Although strong temperature gradients occur near the walls, the chosen mesh resolution must also be sufficient to resolve the smallest bubbles and their interfaces throughout the domain. A graded mesh with coarser resolution in the bulk would reduce the ability to capture bubble deformation and coalescence, while refining both wall and bulk regions would exceed the available computational budget. Therefore, a uniformly fine mesh was selected as a compromise to adequately resolve both wall gradients and bubble-scale phenomena.

The planar 2D treatment with empty patches is standard in OpenFOAM when no through-width physics is modelled. Boundary patches are named left (inlet), right (outlet), top/bottom (walls), and front/back (empty).

### 2.4. Phases and Thermophysical Models

Of the two phases considered, the gas phase is treated as a perfect gas, and the ideal-gas equation of state (EOS) is used to model the frequently used blowing agents nitrogen and carbon dioxide. The gas viscosity and gas Prandtl number are chosen as constant parameters because the temperature dependency is negligible in the temperature and pressure range of the foam injection moulding process. The relevant domain ranges from the melt temperature to cell stabilisation.

The plastic melt’s density (ρ_m_) is given by a reciprocal polynomial EOS, where $C_{i}$ are the polynomial coefficients of any order (N):

(2) $$\frac{1}{\rho_{m}}=C_{0}+C_{1}T+C_{2}T^{2}-C_{3}p-C_{4}pT$$

This compact form captures the dominant thermal expansion with a quadratic temperature dependence and allows for a modest pressure coupling. As the focus was on the filling and foaming regime rather than precise packing or warpage prediction, this available function in OpenFOAM was used instead of implementing a full Tait law [27].

The viscoelastic behaviour of the polymer melt is described using the built-in Phan–Thien–Tanner (PTT) model available in OpenFOAM v11. In this implementation, the polymeric extra-stress tensor is solved as an additional transport equation and incorporated directly into the momentum equation, replacing the turbulent stress contribution. Thus, the solver operates as a viscoelastic compressible VoF formulation without the use of external rheology libraries. The melt behaves as a shear-thinning, viscoelastic fluid. We therefore model μ(T,$\dot{\gamma }$) and the elastic stresses with a multi-mode Phan–Thien–Tanner (PTT) constitutive law, so that the simulation captures both the viscous and history-dependent (elastic) contributions to stress. PTT is a network-based model that reproduces shear-thinning and normal stress differences and provides a finite, rate-dependent extensional viscosity in uniaxial/planar/biaxial extension; depending on the parameters, it can also exhibit pronounced strain-/rate-dependent extensional thinning, which is relevant when the material is strongly stretched.

For foaming flows, it is useful to distinguish four stress contributions: (i) viscous shear stresses, (ii) elastic (transient) shear stresses, (iii) viscous extensional/normal stresses, and (iv) elastic (transient) extensional/normal stresses. The extensional/normal components are especially important here because cell growth imposes predominantly biaxial extension at the bubble wall, and bubble growth predictions have been shown to depend strongly on the transient extensional viscosity rather than the shear viscosity alone [28,29,30,31]:

(3) $$\frac{\delta \tau }{\delta t}+\nabla \cdot \left(U\tau \right)-2symm\left[\tau \cdot \nabla U\right]=-2\frac{\upsilon_{M}}{\lambda }symm\left(\nabla U\right)-\frac{1}{\lambda }exp\left(-\frac{\varepsilon \lambda }{\upsilon_{M}}tr\left(\tau \right)\right)\tau$$

In Equation (3), τ denotes the polymeric extra-stress tensor, U the velocity, and ∇U the velocity gradient tensor. The operator symm(.) extracts the symmetric part of a tensor, and tr(τ) denotes the trace. The material parameters are the polymeric viscosity scale ($\upsilon_{M}$), the relaxation time (λ) and the nonlinearity parameter (ε). The left-hand side ∂τ/∂t + ∇ (Uτ) represents the unsteady transport of polymer stress with the flow, while the term 2 symm(τ ∇U) provides the frame-invariant convected kinematics typical of differential viscoelastic models.

The term (2νM/λ) symm(∇U) acts as the deformation-driven stress source, and the exponential factor exp[−(ελ/νM)tr(τ)] introduces the characteristic PTT network damping, which regularises stress growth and yields a finite, rate-dependent extensional response. PTT has also been used in polymer foaming cell growth simulations, supporting its relevance for the micro-mechanics of foaming [30].

The heat capacity and thermal conductivity of the melt were set as temperature-independent for this work. However, tabulated temperature-dependent values can be implemented in this model if required. The melt viscosity parameter ($\upsilon_{M}$) is defined using temperature-dependent tabulated values.

The initial condition is a fully liquid channel (φ = 0 everywhere). Gas nuclei and their distribution arise from the nucleation model described in Section 3. The VoF transport herein simply carries the gas fraction once present. However, the pressure field inside the bubbles is not driven by diffusion from the solved gas in the melt towards the nucleated bubbles, as it is not part of the compressibleVoF solver. The gas concentration cannot be used as a state variable, and the cell growth kinetics is thus described with a substitution model (see Section 3.5).

### 2.5. Boundary and Initial Conditions

Both the inlet and outlet are prescribed with time-dependent pressure boundary conditions derived from macro-scale simulation results. No velocity or mass flux is imposed; instead, the flow is driven entirely by the resulting pressure gradients. The velocity field therefore develops self-consistently from the governing equations under physically consistent boundary conditions. Temperatures at the inlet are set with zero gradient (convective inflow). The top and bottom walls are no-slip for velocity, and the walls’ thermal conditions are imposed from the macro-model as the time-dependent wall temperature.

### 2.6. Temporal and Algorithmic Settings

The time integration uses an adaptive time step controlled by both the flow Courant number and the VoF Courant number: maxCo = 0.25 and maxAlphaCo = 0.25. These bounds are typical for VoF with explicit interface compression and help avoid aliasing of capillary waves without explicitly computing a capillary time-step limit.

The PIMPLE settings were nOuterCorrectors = 3, nCorrectors = 3, and nNonOrthogonalCorrectors = 0. Interface compression employed the OpenFOAM interface compression scheme with a compression coefficient of 1 and MULES bounding. Discretisation used second-order schemes in space and a first-order Euler in the time scheme.

### 2.7. Surface Tension and Capillary Treatment

Surface tension was included through the CSF model of Brackbill et al., where curvature is obtained from the phase fraction field. This formulation is the default in OpenFOAM’s VoF solvers [32]. While various geometric VoF curvature options exist, we retained the standard algebraic CSF/VoF for consistency with the intended multiscale workflow. To promote stability with explicit surface tension and interface compression, we limited maxAlphaCo, as stated above; no additional capillary time-step constraint was enforced. While geometric VoF approaches can provide improved interface curvature estimation and reduce spurious currents, their implementation for compressible, viscoelastic two-phase flows is currently limited. Therefore, the standard CSF formulation available in OpenFOAM v11 was employed to enable a consistent multiscale framework. The focus of this study is on the coupling of bubble nucleation, growth representation, and flow interaction rather than on highly accurate curvature reconstruction.

## 3. Bubble Nucleation

**Figure 1:**
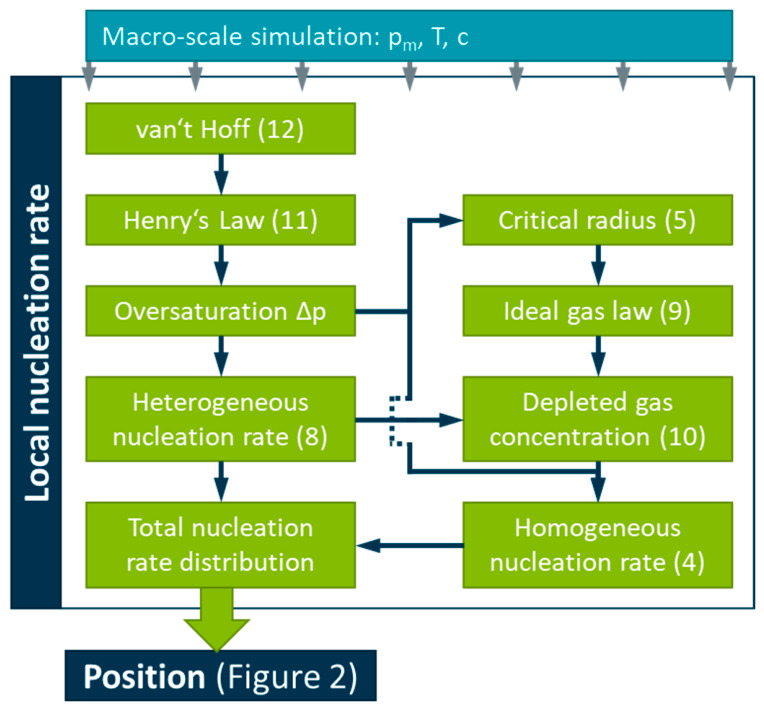
The process of determining the local nucleation rate with the pressure, temperature and gas concentration distribution as input before progressing to the next steps in Figure 2.

In the present work, the initial bubble population for the micro-scale foam injection moulding simulations was generated in a pre-processing step from a physically based nucleation model (Figure 1). It was programmed with Python 3.11.9 and imported the pressure and temperature results of the OpenFOAM simulation as input parameters. The goal was to obtain a spatially inhomogeneous distribution of bubble centres, radii and internal pressures that was consistent with the local thermodynamic state of the polymer melt and could be used as the initial condition for the two-phase flow solver.

### 3.1. Local Nucleation Rate

Nucleation is treated as a stochastic process described by classical nucleation theory (CNT) [33,34,35]. The local homogeneous nucleation rate (N_hom_) describing the number of critical nuclei formed per unit volume and time is taken as:

(4) $$N_{\mathrm{hom}}\;=\;c_{\mathrm{hom}}\cdot f_{1}\cdot exp\left\{-\frac{∆G_{hom}^{*}}{k_{B}\cdot T}\right\}$$

where c_hom_ is the local gas concentration during the homogeneous nucleation, $k_{B}$ is the Boltzmann constant, and T is the local melt temperature. The factor f_1_ is a frequency factor representing the attempt frequency for molecular attachment to a critical nucleus. For gas bubble nucleation in a liquid, the critical free energy ($∆G_{hom}^{*}$) barrier is the maximum of the excess Gibbs free energy. The maximum is reached at the critical radius (r*), which is defined as follows [34,35]:

(5) $$r^{*}=\;\frac{2\sigma }{\Delta p}$$

where σ is the polymer–gas surface tension and Δp is the effective pressure difference between the gas in the nucleus and the surrounding polymer melt. The corresponding critical Gibbs free energy barrier for a homogeneous nucleation is:

(6) $$∆G_{hom}^{*}=\;\frac{16\;\pi \;\cdot \sigma^{3}}{3\cdot \Delta p^{2}}$$

For a given Δp, only nuclei with r > R* are thermodynamically supercritical and can grow. Subcritical nuclei r < R* tend to dissolve. CNT relates the nucleation rate to this barrier via a Boltzmann factor (exp(−ΔG*/k_B_T)) in Equation (4) [34,35]. However, in gas-loaded polymer melts, nucleation is known to be predominantly heterogeneous at interfaces or impurities like interfaces, additives, fillers or other impurities in the solution, whereas homogeneous nucleation is strongly suppressed by the large free energy barrier [36].

For heterogeneous nucleation on a substrate, the critical nucleus has the shape of a spherical cap rather than a full sphere. The corresponding free energy barrier is reduced by a geometric factor (f(θ)) that depends on the contact angle (θ) between the nucleus and substrate [34,35]. Therefore, the critical Gibbs energy for heterogeneous nucleation is reduced by this factor:

(7) $$∆G_{het}^{*}=f(\theta )\cdot ∆G_{hom}^{*},\;\;\;\;\;f(\theta )=\frac{1}{4}(2+cos\theta ){\left(1-cos\theta \right)}^{2}$$

The $∆G_{hom}^{*}$ is the homogeneous barrier defined above (Equation (4)). For 0 < θ < π, 0 < f(θ) < 1, so heterogeneous nucleation is energetically favoured. In the limit θ → 0, the barrier can be reduced by several orders of magnitude. The implemented heterogeneous nucleation rate is:

(8) $$N_{\mathrm{het}}\;=\;c_{\mathrm{het}}\cdot f_{2}\cdot exp\left\{-\frac{∆G_{hom}^{*}}{k_{B}\cdot T}\right\}$$

Both kinds of nucleation are summed in the script to determine a total nucleation rate. However, because the heterogeneous nucleation is energetically favoured, it is assumed that it occurs first and consumes a portion of the dissolved gas before homogeneous nucleation becomes relevant.

### 3.2. Gas Depletion by Heterogeneous Nucleation

The depletion of supersaturation by earlier nuclei is a standard effect in CNT and in the kinetics of phase transformation [35,36]. To determine the reduced gas concentration, the amount of gas in a critical cell nucleus is calculated with the ideal-gas law:

(9) nhet∗=pm·34π·r∗R·T

The local melt pressure ($p_{m}$) is thereby assumed for the critical gas nucleus. Consequently, the reduced concentration for the homogeneous nucleation ($c_{hom}$ [mol/m^3^]) is calculated with:

(10) $$c_{hom}=c_{het}-\frac{N_{het}\cdot \Delta t_{nuc}\cdot n_{het}^{*}}{N_{A}}$$

Here, the nucleation density is calculated with the nucleation time ($\Delta t_{nuc}$) and divided by the Avogadro constant ($N_{A}$). The result cannot be negative, and if the gas depletion by heterogeneous nucleation is overestimated, the $c_{hom}$ is set to 0.

### 3.3. Driving Pressure Difference

For a gas dissolved in a polymer melt, the thermodynamic driving force for nucleation is determined by the supersaturation of the dissolved gas relative to its equilibrium solubility at the local pressure and temperature [37,38,39,40]. The script describes gas solubility by Henry’s law:

(11) $$c_{eq}=k_{H}(T)\cdot p_{eq}$$

where $c_{eq}$ is the equilibrium gas concentration in the polymer, $p_{m}$ is the saturation pressure and k_H_(T) is the Henry coefficient, which depends on temperature. The temperature dependence of k_H_ is modelled using a van’t Hoff-type relation, where $k_{H,ref}$ is the Henry coefficient at a reference temperature ($T_{ref}$), $∆H_{S}$ is the enthalpy of the solution of the gas in the polymer and R is the universal gas constant [38,40]:

(12) $$k_{H}\left(T\right)=k_{H,ref}\cdot exp\left[-\frac{∆H_{S}}{R}\left(\frac{1}{T}-\frac{1}{T_{ref}}\right)\right]$$

With Equations (11) and (12), the partial pressure of the gas is used to calculate its difference towards the local pressure of the oversaturated melt. Thus, the homogeneous nucleation rate can be calculated depending on the local pressure and temperature field.

### 3.4. Stochastic Placement of Bubbles

**Figure 2:**
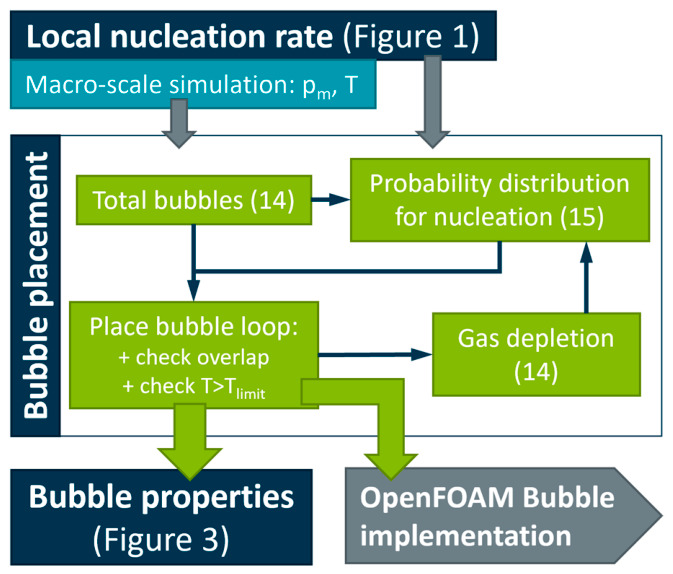
The process of determining the bubble position with the local nucleation rate, temperature and melt pressure distribution as input.

The flowchart of the bubble placement algorithm is displayed in Figure 2, and the used calculations are explained as follows. Nucleation events are assumed to be statistically independent and rare in space–time and are therefore modelled as a non-homogeneous Poisson point process with intensity (N_tot_) [15,36,41,42]. Over a finite nucleation time window (Δt_nuc_), the expected number of nuclei in cell i with its cell volume ($V_{i}$) is:

(13) $$z_{i}=N_{tot,i}\cdot V_{i}\cdot \Delta t_{nuc}$$

On the whole domain, the expected total number of nuclei is:

(14) $$Z=\sum_{i}z_{i}=\Delta t_{nuc}\sum_{i}N_{i}\cdot V_{i}$$

The probability that a nucleation event occurs in a small volume element ($V_{i}$) during a short time interval ($\Delta t_{nuc}$) is then $z_{i}$. For Monte Carlo sampling, this corresponds to an inhomogeneous Poisson point process [43]. This formulation implies that bubble positions are sampled from a spatially varying Poisson point process, where the local nucleation rate defines the placement probability. Discretising the domain into finite volumes with local nucleation rates and volumes, we obtain a spatial probability distribution:

(15) $$\omega_{i}=\frac{J_{i}V_{i}}{\sum_{j}J_{i}V_{i}}=\frac{z_{i}}{Z}$$

Next, the algorithm starts a loop with the iteration n = {1, Z}. The final placement of the bubbles is conducted by selecting a cell (i) according to the nucleation probability distribution. In each iteration, it is checked whether a significant bubble size can be reached by checking whether the local temperature (T) is above a limit temperature (T_limit_). This results in nucleations that do not result in an actual bubble placement but trigger the next iteration. Additionally, a geometric overlap check with existing bubbles is performed, and the iteration repeats if a newly placed bubble intersects with previously placed ones.

For each placed bubble, the local gas concentration is depleted, leading to a reduction in the nucleation probability in its vicinity and thereby suppressing the clustering and unphysical overlap of bubbles. The reduction depends on the gas depletion factor (F) and the depletion range multiplier (G). The local nucleation probability inside the bubble radius ($r_{bubble}$) reduces by the whole factor (F), and outside the bubble, the effective depletion factor decreases linearly. Thus, bubble positions are generated via a non-homogeneous Poisson point process with additional physical gas depletion and geometric overlap check constraints to ensure a physically plausible, non-overlapping distribution:

(16) $$\omega_{i,new}\left\{\begin{array}{c} r\leq \;r_{bubble}:\;\omega_{i,old}\cdot F\;\\ r>\;r_{bubble}:\;\omega_{i,old}\cdot F\cdot \left[1-\frac{r-r_{bubble}}{G{\cdot r}_{bubble}-r_{bubble}}\right] \end{array}\right.$$

### 3.5. Initial Cell Properties and Emulation of Diffusion-Controlled Growth

**Figure 3:**
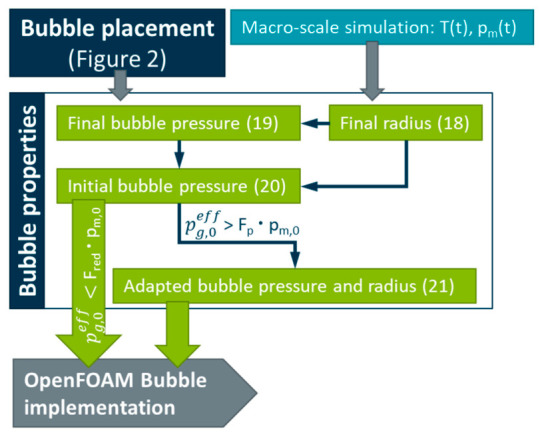
The process of determining the bubble properties with the bubble location, local melt pressure and temperature over time as input.

The following approach is introduced as a temporary surrogate to enable bubble expansion within the current modelling framework (Figure 3). It is not intended to provide a physically accurate description of diffusion-controlled growth but rather to allow for a qualitative investigation of bubble–flow interaction in the absence of an explicit mass-transfer model.

For the initialisation in OpenFOAM, the initial radius, pressure and temperature for each cell need to be defined. The bubble temperature (T_bub,i_) is assumed to be the same as the local melt temperature (T_m,i_). The radius of the bubble is then determined as the critical radius (r*). However, in the real foaming process, bubbles nucleate at nanometre-to-sub-micrometre critical radii and grow by diffusion of dissolved gas into the bubble, while the polymer is still sufficiently mobile [44]. Therefore, such small bubbles cannot be resolved by the computational mesh, and a larger initial radius must be prescribed. Furthermore, cell growth arrests locally once the polymer cools and solidifies either before or after reaching a depictable size. Consequently, one criterion for whether a bubble can be initialised is that the smallest depictable radius (r_min_) is reached until bubble stabilisation. Therefore, a temperature limit (T_limit_) 20 K above the no-flow temperature is used as a nucleation criterion. The value of 20 K is currently a fitting parameter that reflects the cooling that occurs during the cell’s growth phase, which is necessary for it to reach a significant size. If the temperature at the position chosen by the probability distribution is below the limit, the current iteration of the loop is skipped.

The next criteria of the initial radius results from the substitute model for cell growth kinetics. As explained in Section 2.4, the CFD model is currently lacking a physical cell growth model due to the mass in a bubble being constant and no description of the gas diffusion. Therefore, a surrogate cell growth model assigns higher initial pressure instead of increasing the pressure over time. This forces the bubbles to grow during the simulation. The term ‘surrogate’ is used here to emphasise that the model does not calculate physical mass transfer but mimics its macroscopic effect on bubble expansion. But as a consequence, the cell growth is faster than in reality, and the initial pressure difference between the bubble and the melt can lead to divergence in the CFD simulation. Thus, the initial bubble radius and pressure are determined by the model described as follows.

The aim is to choose an effective initial gas pressure ($p_{g,0}^{eff}$) for an initial radius (r_ini_) in a way that, under the process pressure and temperature history, the bubble volume evolution approximates the desired change from r_0_ to the final bubble radius r_f_. For diffusion-controlled cell growth kinetics in a supersaturated liquid, classic analyses by *Epstein and Plesset* and subsequent extensions to polymer melts show that, over a wide range of times, the bubble radius satisfies:

(17) $$r{\left(t\right)}^{2}-r_{0}^{2}\approx 2Kt_{grow},$$

where r_0_ is the initial radius, $t_{grow}$ is the growth time, and K is a growth constant that depends on the gas diffusivity, solubility and supersaturation [45,46]. Neglecting r_0_ compared to the final radius implies:

(18) $$r\left(t\right)\propto \sqrt{t_{grow}}$$

**Figure 4:**
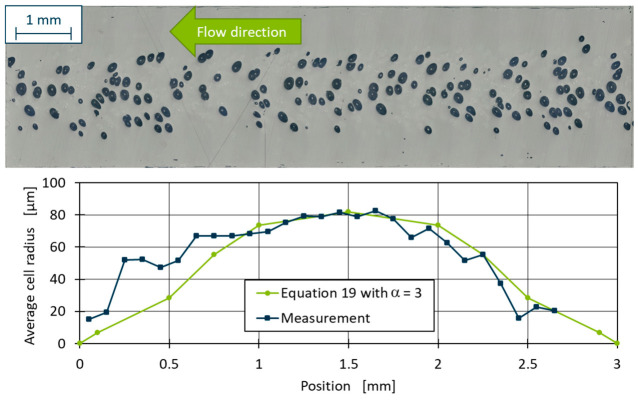
An exemplary microscopy image of a section cut from a N_2_ foamed PC part and its average cell radius distribution together with the fit (Equation (19)).

In a non-isothermal injection moulding process, the available growth time is limited by the local freezing of the polymer. So, the growth time is the difference between the start of the nucleation (t_nuc_) and the time (t_freeze_) at which the plastic melt drops below the above-described limit temperature (T_limit_). This growth time distribution is derived from the macro-scale. Except for areas with T(t_nuc_) < T_limit_, the final radius (r_f_) can be described as:

(19) $$r_{f}=r_{max}{\left(\frac{t_{grow}}{t_{grow,max}}\right)}^{\alpha }$$

where r_max_ is a reference radius taken from a measured cell radius in the core region or from the macro-scale simulation, t_grow,max_ is the maximum growth time distribution and α ≈ 1/2 is consistent with diffusion-controlled growth [45,46]. However, α is regarded as a calibration parameter. A comparison with measured cell size gradients across the thickness, like in Figure 4, shows a better fit with α = 3. This is displayed in Figure 4 for PC and nitrogen (N_2_) as an exemplary material combination.

After the final radius, the final pressure state ($p_{g,f}$) is matched by assuming mechanical equilibrium between the gas and melt at the end of the growth period:

(20) $$p_{g,f}\approx p_{m,f}+\frac{2\sigma }{r_{f}}$$

where $p_{m,f}$ is the local melt pressure at the final time, and 2σ/$r_{f}$ is the Laplace pressure contribution, which is only significant for tens-of-micrometre bubbles at MPa-level pressures.

Afterwards, the ideal-gas law together with the same effective gas amount during initialisation ($n_{0}^{eff}$) and after cell stabilisation ($n_{f}^{eff}$) allows for the calculation of the initial pressure for each bubble:

(21) $$p_{g,0}^{eff}=pg,f{\left(\frac{r_{f}}{r_{o}}\right)}^{3}*\frac{T_{0}}{T_{limit}}$$

Depending on the minimum radius, the initial pressure for larger bubbles in the centre can be multiple times higher than the local melt pressure. This results in divergence in the CFD simulation. Therefore, a reduction in these initial pressures with the same amount of gas is necessary. This is realised by increasing the initial radius of all bubbles with $p_{g,0}^{eff}>$ $p_{m,0}\cdot F_{p}$. $F_{p}$ is the factor of overpressure, which is usable by the CFD simulation and is defined by tests. The new initial radius ($r_{0,\mathrm{new}}$) with the same gas amount is calculated with the ideal-gas law:

(22) $$r_{0,\mathrm{new}}=\;r_{0,\mathrm{old}}{\left(\frac{p_{g,0}^{eff}}{p_{g,0,neu}^{eff}}\right)}^{1/3}\mathrm{with}\;p_{g,0,neu}^{eff}=p_{m,0}\cdot F_{p}$$

In other words, all gas that would have diffused into the bubble during microscopic growth from r_0_ to r_f_ is effectively assigned at the beginning of the CFD simulation at the numerically resolvable radius (r_0_). The resulting larger gas mass causes the bubble to expand under the subsequent pressure drop. The expansion is slowed down by the inertia and resistance of the melt viscosity in a way that approximates the desired effective growth.

While this approach does not reproduce the detailed kinetics of diffusion-controlled growth, it provides a physically interpretable way to initialise bubble sizes and internal pressures in a two-phase flow simulation without explicit mass transfer, consistent with classical bubble growth theory and with observed variations in the final cell size across the moulded part [44,45].

An optional distance check to avoid overlapping bubbles can also be activated. However, the gas depletion (Equation (16)) already reduces this probability depending on the chosen depletion parameters.

## 4. Demonstration Case

### Simulation Setup

**Figure 5:**
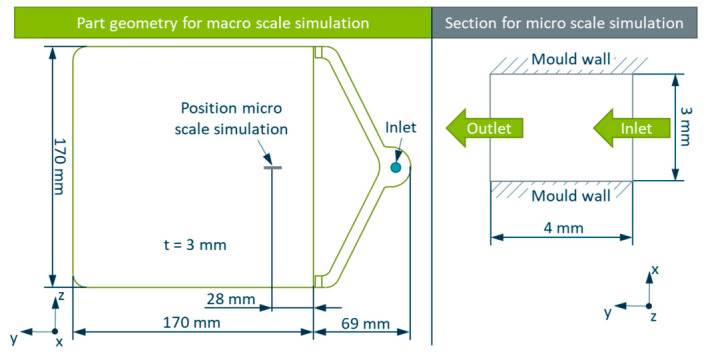
The part geometry, position and size of the micro-scale simulation.

To demonstrate the two-phase CFD simulation, an injection moulded part with a simple plate geometry was used (Figure 5). The mould of the part has a cold runner that distributes the melt over the whole plate width and ensures a uniform flow front. The modelled materials were the PC LED 5102, Covestro AG, Leverkusen, Germany, and nitrogen gas. Their material and model parameters are listed in Table 1 and Table 2. The zero-shear viscosity is defined as:

(23) $$\eta_{0}=D_{1}\cdot exp\left(\frac{-A_{1}(T-T_{ref})}{A_{2}(T-T_{ref})}\right)$$

with the reference temperature (T_ref_) and the fitted coefficients A_1_, A_2_ and D_1_. The parameters of the van’t Hoff equation are approximated from literature values [4,47].

**Figure 6:**
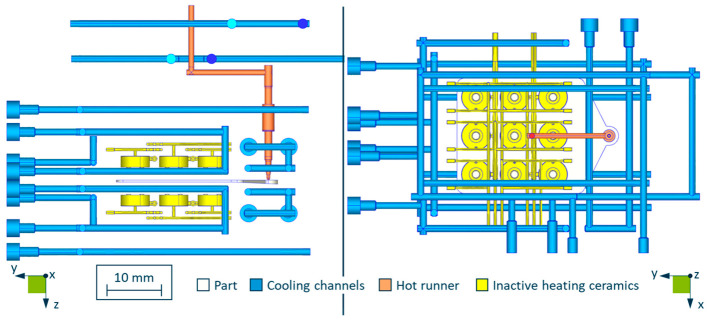
The process setup of the macro-simulation with cooling channels and the unused heating ceramics of the fixed mould half.

The boundary conditions of the micro-scale simulation, like the wall temperature and pressure development, were derived from a macro-scale simulation. It was conducted with the injection moulding simulation software Moldex3D 2024, CoreTech System Co., Ltd., Zhubei, Taiwan, and its foam injection moulding module (Figure 6). The process parameters are displayed in Table 3. The cooling channels were considered, and a transient cooling simulation was calculated. Due to the different available material models, the macro-simulation was conducted with a Tait model and modified cross-WLF model instead.

The micro-scale simulation calculated the flow in a 4 mm long section in the centre of the plate (Figure 5). It started with a pure melt flow until the injection phase stopped at 1.05 s, the pressure dropped suddenly and the gas nucleated [49]. Three different nucleation times were investigated to consider the time required for the bubbles to reach a mesh-resolvable size. Nucleation also happens in the filling stage but mostly happens at the flow front where the pressure is low. After the simulation of the filling stage, the pressure and temperature field of the micro-scale simulation were imported into the nucleation script. This was not done with the state variables calculated by the macro-simulation because its mesh resolution is lower and additional interpolations would be necessary to map the results on the micro-simulation mesh. The nucleation script was run with the parameters in Table 4. Three different bubble distributions with different overpressure factors (F_p_) (see Equation (22)), 1.0, 1.5 and 2.0, were generated to analyse their effects. Afterwards, the determined bubble distribution and the bubbles’ properties were implemented as the second phase of the CFD simulation.

The graphs showing the results of the nucleation algorithm were created with a Python script written by the generative artificial intelligence Claude Haiku 4.5. It used the data of the nucleation algorithm and OpenFOAM simulation as input.

## 5. Results and Discussion

### 5.1. Macro-Scale Simulation

**Figure 7:**
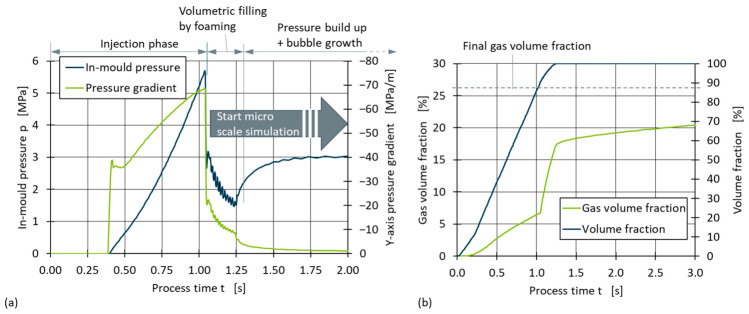
The pressure and pressure gradient along the flow direction development of the macro-scale simulation at the position of the micro-scale simulation domain (**a**) and the development of the gas volume and volume fraction (**b**).

The macro-simulation showed a typical pressure development for a short shot (95 of 109 cm^3^) in a low-pressure foam injection moulding process. Figure 7a displays the increasing pressure in the filling phase and the sudden pressure drop afterwards, which is measured at the position of the micro-scale simulation (Figure 5). This was followed by a pressure decline until the cavity was filled completely. The pressure gradient in the direction of the flow (y-axis) behaved similarly, as it also dropped, and the decompression as well as the remaining volume flow due to foaming led to a declining gradient. Both graphs show oscillating curves during the foaming right after the injection phase because the simulation cannot depict the pressure development at this high foaming rate.

This foaming rate increased significantly due to the pressure drop, and the average gas volume fraction rate changed from 8.0%/s during the filling stage to 53.7%/s. When the remaining 13% of the cavity was filled after ca. 0.2 s, the cavity pressure rose again due to continued foaming, and the foaming rate decreased again (Figure 7). However, the simulation calculated a remaining pressure gradient and volume flow after that. This is explained by the different foaming and the resulting lower density at the flow path end.

### 5.2. Nucleation

**Figure 8:**
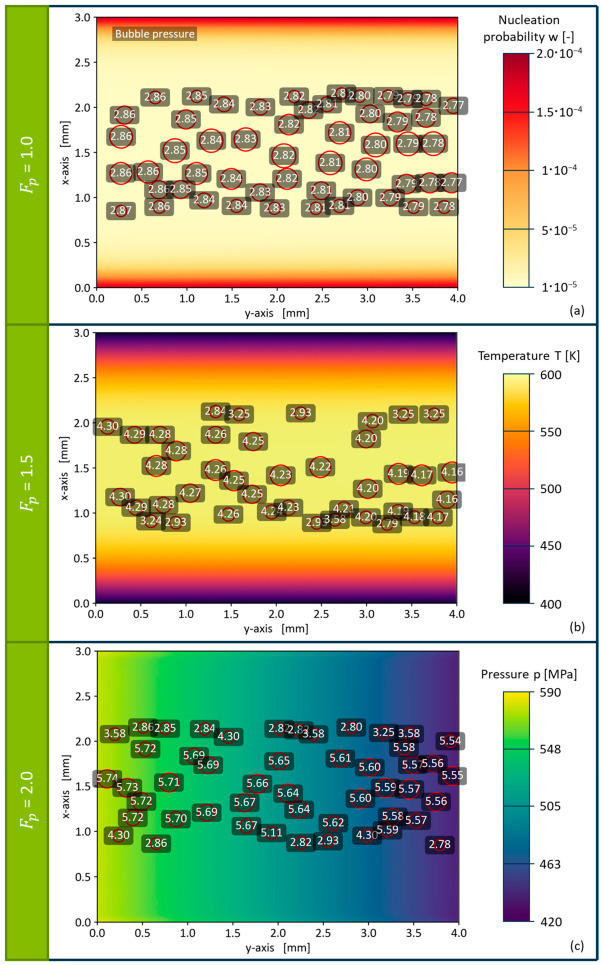
The nucleated bubble distributions (red circles) with the overpressure factors 1.0, 1.5 and 2.0, including the resulting initial bubble pressures, as well as the common initial distributions of the nucleation probability (**a**), temperature (**b**) and pressure (**c**).

As major nucleation and cell growth happen due to and after the pressure drop, modelling the bubbles in the micro-scale after this time is a justified simplification. Even later timings for the implementation of the bubbles can be justified by the nucleation duration and the time required for the bubbles to reach a size that can be discretised by the mesh. Consequently, the pressure and temperature distribution were used for three nucleation cases. The resulting bubble distributions for overpressure factors of 1.0, 1.5 and 2.0 are displayed in Figure 8. The distribution shows bubbles in the centre of the flow channel, as the bubbles at the edge layer were too small for the mesh to depict them.

The bubbles that grow big enough have the highest radii in the centre according to Equation (19), and when F_p_ > 1, their radii are reduced and the initial bubble pressure in the centre is increased. In contrast, the bubbles closer to the edge are less affected by the F_p_ and pressure, and the radii are similar for all three distributions because their size cannot be decreased below the radius limit.

The nucleation events, according to the calculated nucleation probability (15), happen mostly in the edges, as the probability is higher in the cooler regions due to the temperature dependency of the Henry coefficient (Figure 8) [50,51]. Consequently, of around 1000 nucleation events, around 50 results in an actual bubble placement for the micro-scale simulation. Inside this core layer, the nucleation algorithm calculates a reasonable distribution suitable for a first application of the micro-scale simulation model. The defined bubble size distribution displayed in Figure 4 shows similar-sized bubbles with a decreasing bubble size towards the surface layer. The initial distribution therefore agrees qualitatively with the experimental observations.

For a quantitative application, the parameters need to be determined with high certainty, as the nucleation is very sensitive towards them and the literature values vary significantly [4,47,52]. Additionally, one iteration of the nucleation algorithm results in incomplete bubble distribution, as, in reality, smaller bubbles that nucleate later and have less time to grow also appear between the bigger bubbles. This can be modelled by stopping the micro-scale simulation every time a new iteration nucleation should be performed and adding the additional bubbles before continuing the simulation.

### 5.3. Micro-Scale Simulation

**Figure 9:**
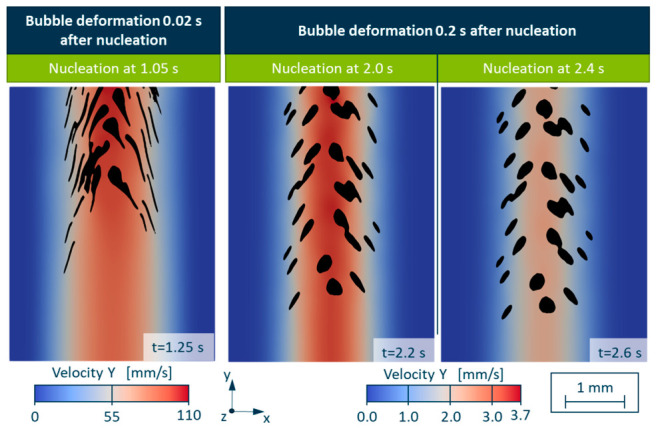
Simulated bubble shape (black) and velocity field with different nucleation times.

The determined bubble distributions (Section 5.2) and pressure development (Section 5.1) with nucleation at 1.05 s resulted in the cell growth and deformation depicted in Figure 9. At 0 s after the nucleation, the bubbles were placed in a laminar flow, which already induced the early deformation of the bubbles due to shearing. The bubble deformation due to shearing depicted in Figure 9 0.02 s after the initialisation of the bubbles is more pronounced than expected. This could have resulted from the too-high pressure gradients and melt inertia calculated by the macro-scale simulation, as well as from the big initial bubble size. A high initial radius subjects the bubbles to higher shearing because the velocity difference of both sides of the bubble is higher. Additionally, a round shape is more supported by the surface tension for smaller radii, and deformation by outer forces like shear stress occurs when they dominate the surface tension. This was not the case for this initial large bubble size.

The simulation results in Figure 9 show the bubble deformation after 0.2 s of shear flow, when the bubbles were initialised at 2.0 s and 2.4 s instead of at 1.05 s. In this investigation, the previous bubble deformation was neglected. The pressure gradient in the y-direction decreased from 10.5 MPa/m at 1.05 s to 1.1 MPa/m at 2.0 s and 0.9 MPa/m at 2.4 s. Because of the greater pressure difference and the melt inertia of the flow right after the injection stop, the maximum flow velocity was 46 times higher at 1.05 s than at 2 s.

This resulted in the lower shearing and slower deformation of the bubbles, which have a more realistic shape. However, before stabilisation of the bubbles and freezing of the melt, the deformation continued, and the deformation exceeded the bubble shape found in practice, which is displayed in Figure 4 for the same plastic and process conditions. Since an even later nucleation is physically unlikely, it can be concluded that the pressure gradient during the foaming phase calculated by the macro-scale simulation needs to match with reality more precisely.

**Figure 10:**
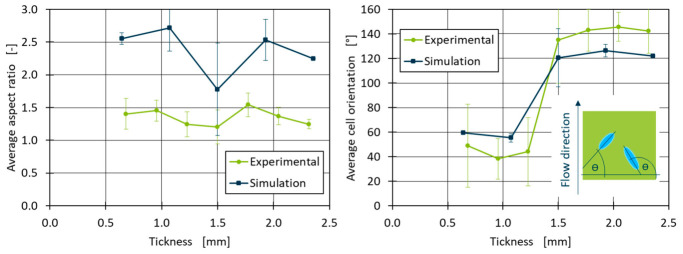
Qualitative comparison of experimental and simulated (nucleation at 2.4 s) cell aspect ratios and orientations with blue gas cells and green matrix.

For the nucleation time of 2.4 s, Figure 10 compares the simulated and experimentally measured cell aspect ratios and orientation distributions across the part thickness. The aspect ratio and orientation were evaluated using ImageJ (version 1.54d) and the plugin EllipseSplit and compared with the reflected-light microscopy measurements shown in Figure 4 [53]. The previously discussed exceeding deformation is shown by the higher aspect ratio of the simulation results. The higher deformation history resulted in the longer bubble shapes. However, the course of the aspect ratio matched the characteristic cell geometry of the FIM process. The minimum aspect ratio was in the centre, where the shearing was the lowest, and it increased in the shearing zone before decreasing due to smaller bubbles and earlier freezing.

The orientation comparison also shows similar distributions across the part’s thickness in the simulation and experiments. The orientation is at its maximum in the shear zone and decreases towards the edges. The average orientation in the centre of the part was higher than expected in both the simulation and experiment, where values close to 90° were predicted. This resulted from the high range of orientation values with a standard deviation peak of 23.7° for the simulation. The lower aspect ratio and less shear-dominated deformation of the bubbles in the centre led to a deformation that was dominated by the bubble interactions. Small changes in shape due to other bubbles being close resulted in a significant change in the orientation. This observation is also the reason for the higher standard deviation of the experimental orientation, which was more prone to variations due to its low aspect ratio.

This agreement indicates that the proposed micro-scale framework is capable of capturing the key mechanisms governing bubble deformation and orientation under realistic processing conditions. In particular, the model reproduces the characteristic skin–core morphology with respect to aspect ratio and orientation distribution, which is not accessible with conventional spherical-cell approaches.

For cases with elevated overpressures (F_p_ = 1.5 and 2.0), bubbles located directly at the inlet and outlet had to be removed, as they caused divergence in the pressure calculation. This behaviour is attributed to the combination of strong local pressure gradients, the later-explained immediate oscillation of overpressured bubbles, and backflow at the domain boundaries. This intervention was only required for the surrogate growth cases with imposed overpressures and not for cases without overpressure.

The influence of removing these boundary-contacting bubbles on the global flow behaviour and qualitative trends was found to be limited. The affected bubbles represent only a small fraction of the total gas phase, and the surrounding melt flow rapidly fills the local region. Therefore, the overall flow dynamics and bubble evolution remain qualitatively consistent, and the results provide a basis for analysing bubble–flow interaction.

**Figure 11:**
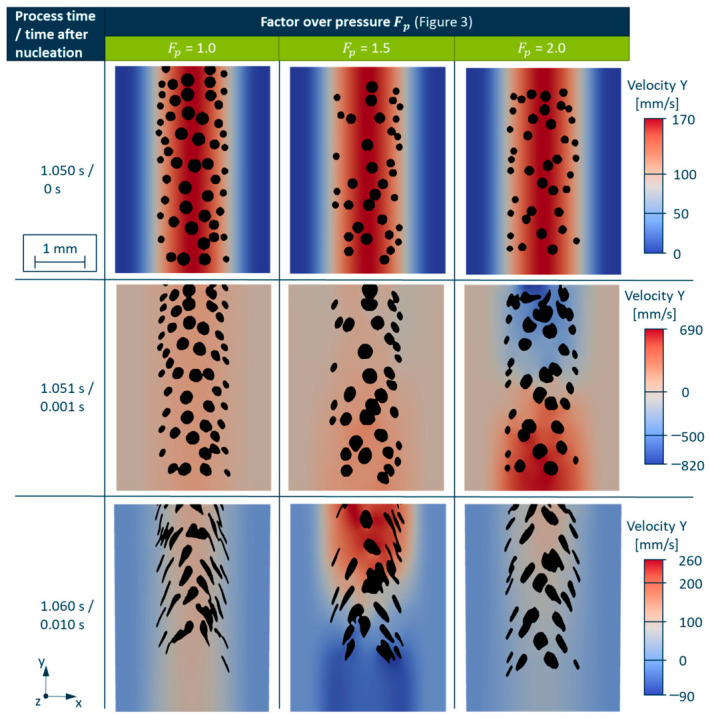
The simulated bubble shape (black) over time with overpressure factors of 1, 1.5, and 2.0, including the resulting velocity distribution.

This intervention does not affect the underlying modelling approach for nucleation and bubble deformation. Consequently, the framework remains suitable as a proof of concept for multiscale coupling despite this limitation. The reason for the divergence became apparent in the subsequent time steps. While the area of bubbles with no overpressure did not grow, the other two cases showed a sudden increase in the bubble size at 0.001 s (Figure 11). This indicates that the viscosity of the melt slowed down the pressure equalisation and cell growth only slightly. As a result, the fast cell growth influenced the local melt flow velocity significantly. For the factors 1.5 and 2.0, the velocity increased to several times the initial speed at the outlet (x = 4) and became negative close to the inlet (x = 0) during the bubble expansion. Additionally, an oscillation of the bubble size was observed due to the viscoelastic viscosity model. Consequently, the effect on the melt velocity appeared again the other way around during bubble contraction.

**Figure 12:**
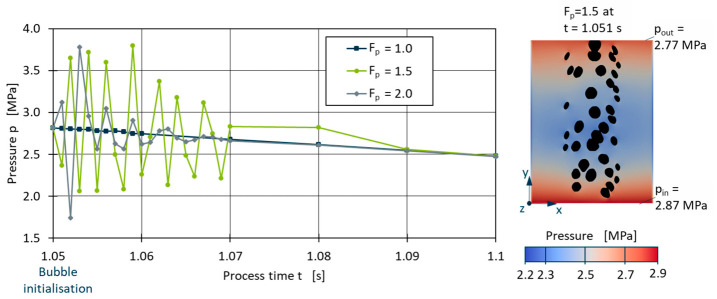
The pressure development in the centre of the micro-scale simulation for different overpressure factors and the pressure field with black gas bubbles shortly after bubble initialisation.

In Figure 12, the pressure in the centre of the regarded flow channel is depicted. The oscillation of the pressure represents the oscillation of the bubble size. The assigned pressure decrease was followed by the simulation with no overpressure, and the expected flow velocity continued monotonously. In contrast, the two simulations with overpressure showed higher and lower pressure than in the inlet and outlet. Even though the oscillation of F_p_ = 2 was higher, it converged towards the regular pressure at around 1.07 s, whereas the lower overpressure converged later at around 1.09 s. This is why in Figure 11 the velocities at 1.06 s for F_p_ = 2 and F_p_ = 1 look similar and F_p_ = 1.5 still has negative volume flow. As there is no significant difference in the total bubble volume and the related total expansion volume between the two overpressure cases, the interaction between the bubbles might have influenced the oscillation.

The observed oscillations are interpreted primarily as a consequence of the surrogate growth strategy rather than as an inherent instability of the VoF–PTT formulation. In the present model, the gas that would physically enter the bubble gradually by diffusion is introduced as an elevated initial bubble pressure at a numerically resolvable radius. This produces an abrupt pressure imbalance and rapid volumetric response on a time scale much shorter than that of physical diffusion-controlled growth. The coupled VoF, surface tension, and viscoelastic stress calculation can amplify this abrupt transient, but the absence of comparable oscillations in the no-overpressure case indicates that the dominant cause is the substitute growth initialisation.

These observations imply that modelling the cell growth kinetics with an initial overpressure is not suitable, as the growth is expected to occur over a longer duration and the unrealistic oscillation affects the resulting bubble shape. Despite these limitations, the two-phase simulation captures key physical mechanisms, such as bubble deformation under shear, interaction between neighbouring bubbles, and coalescence. These phenomena represent the primary contribution of the present micro-scale framework (Figure 9). Accordingly, the presented results are interpreted qualitatively, with emphasis on deformation, orientation, and interaction effects rather than on quantitatively accurate growth kinetics.

## 6. Conclusions

This work presents a micro-scale simulation framework for foam injection moulding that combines a two-phase compressible VoF solver with viscoelastic rheology and a CNT-based nucleation model driven by macro-scale process conditions. The framework enables the simulation of bubble nucleation, deformation, coalescence, and interaction with the surrounding flow field. In a plate geometry case study (polycarbonate with N_2_), the model qualitatively captured these mechanisms in a 2D through-thickness section. The simulated deformation reproduced the characteristic aspect ratio and orientation trends observed in the experiments on a qualitative level.

A simplified surrogate growth approach was used to enable bubble expansion within the current solver framework. The results show that this approximation introduces non-physical oscillations and cannot reproduce realistic growth kinetics. These are interpreted mainly as artifacts of the growth substitution strategy, although their amplitude is amplified by the stiffness of the coupled two-phase viscoelastic CFD problem. The resulting accelerated early growth can exaggerate deformation near the injection stop. Sensitivity to the initial radius and overpressure underscores the need for a physically consistent growth mechanism and careful parameter calibration. Nevertheless, the presented framework provides a proof of concept for multiscale coupling, and its ability to capture essential micro-scale mechanisms governing foam morphology has been demonstrated.

Future work will therefore introduce diffusion-controlled mass transfer to the gas phase to investigate cell growth kinetics without artefacts from the surrogate growth model. The improved model can then be validated against measured through-thickness cell size gradients. Incorporating temperature-dependent thermophysical properties, exploring 3D domains where needed, and tightening the macro–micro-coupling loop are expected to improve quantitative predictiveness for microstructure-aware component design.

## Figures and Tables

**Table 1:** Material parameters of PC and its PTT as well as cross-WLF model parameters for micro-scale simulation.

Material Parameter	Value	Unit
Thermal conductivity	0.23	[W/(m∙K)]
Heat capacity	2000	[J/(kg∙K)]
PTT $\upsilon_{M}$	0.1	[Pa∙s]
PTT λ	0.004	[s]
PTT ε	0.01	[-]
EOS C_0_	6.257 × 10^−4^	[m^3^/kg]
EOS C_1_	5.837 × 10^−7^	[m^3^/(kg∙K)]
EOS C_2_	−3.327 × 10^−12^	[m^3^/(kg∙K^2^)]
EOS C_3_	−8.115 × 10^−13^	[m^3^/(kg∙Pa)]
EOS C_4_	2.987 × 10^−15^	[m^3^/(kg∙Pa∙K)]
Cross-WLF A_1_	7.9837	[-]
Cross-WLF A_2_	162	[K]
Cross-WLF D_1_	1207	[Pa∙s]
Cross-WLF T_ref_	518	[K]

**Table 2:** Material parameters of nitrogen and its solubility parameters in polymer melt.

Material Parameter	Value	Unit
Prandtl number	0.7	[-]
Heat capacity	1045	[J/(kg∙K)]
Molecular weight	28.9	[g/mol]
Surface tension	0.0178	[N/m]
Contact angle [48]	30	[°]
Reference Henry coefficient, k_H_ (298 K) [4,47]	6.5	[mol/(m^3^∙MPa)]
Enthalpy of solution, $∆H_{S}$	10,500	[J/mol]
Viscosity	2.84 × 10^−5^	[Pa∙s]

**Table 3:** Process parameters of macro-scale foam injection moulding simulation.

Process Parameter	Value	Unit
Melt temperature	300	[°C]
Mould temperature	110	[°C]
Volume rate	100	[cm^3^/s]
Gas concentration	0.6	[weight-%]
Switch-over point	95	[vol-%]
Holding pressure time	0	[s]
Cooling time	25	[s]
Mould-opening time	5	[s]

**Table 4:** Model parameters of nucleation algorithm.

Model Parameter	Value	Unit
Maximum bubble radius, r_f_	150	[µm]
Nucleation time, $\Delta t_{nuc}$	0.1	[s]
Gas depletion factor	0.9999	[-]
Depletion range multiplier	4.0	[-]
Minimum bubble radius, r_min_	69	[µm]
Critical growth temperature, $T_{limit}$	450	[K]

## Data Availability

The data and materials for this publication are available upon request at the following link: http://hdl.handle.net/21.11102/e6bc97b7-a112-480b-8545-8870534b2f03 (accessed at 7 July 2026).

## Abbreviations

The following abbreviations are used in this manuscript:

CNT	Classical nucleation theory
CSF	Continuum surface force
DFG	Deutsche Forschungsgemeinschaft
EOS	Equation of state
FIM	Foam injection moulding
LBM	Lattice Boltzmann method
N_2_	Nitrogen
PC	Polycarbonate
VoF	Volume of fluid
